# *QPOLE*: A Quick, Simple, and Cheap Alternative for *POLE* Sequencing in Endometrial Cancer by Multiplex Genotyping Quantitative Polymerase Chain Reaction

**DOI:** 10.1200/GO.22.00384

**Published:** 2023-05-25

**Authors:** Anne Sophie V.M. Van den Heerik, Natalja T. Ter Haar, Lisa Vermij, Jan J. Jobsen, Mariel Brinkhuis, Suzan M. Roothaan, Alicia Leon-Castillo, Gitte Ortoft, Estrid Hogdall, Claus Hogdall, Tom Van Wezel, Ludy C.H.W. Lutgens, Marie A.D. Haverkort, Jas Khattra, Jessica N. McAlpine, Carien L. Creutzberg, Vincent T.H.B.M. Smit, C. Blake Gilks, Nanda Horeweg, Tjalling Bosse

**Affiliations:** ^1^ Radiation Oncology, Leiden University Medical Center (LUMC), Leiden, the Netherlands; ^2^ Pathology, Leiden University Medical Center (LUMC), Leiden, the Netherlands; ^3^ Radiation Oncology, Medisch Spectrum Twente, Enschede, the Netherlands; ^4^ Pathology, Laboratorium Pathologie Oost-Nederland, Hengelo, the Netherlands; ^5^ Department of Gynaecology, Copenhagen University Hospital, Rigshospitalet, Copenhagen, Denmark; ^6^ Department of Pathology, Copenhagen University Hospital, Herlev and Gentofte Hospital, Copenhagen, Denmark; ^7^ Maastricht Radiation Oncology (MAASTRO), Maastricht University Medical Centre+, Maastricht, the Netherlands; ^8^ Radiation Oncology, Radiotherapy Group, Arnhem, the Netherlands; ^9^ Department of Laboratory Medicine and Pathology, Surrey Memorial Hospital, Surrey, BC, Canada; ^10^ Division of Gynecologic Oncology, Department of Obstetrics and Gynaecology, University of British Columbia, Vancouver, BC, Canada; ^11^ Department of Pathology and Laboratory Medicine, University of British Columbia, Vancouver General Hospital (VGH), Vancouver, BC, Canada

## Abstract

**PURPOSE:**

Detection of 11 pathogenic variants in the *POLE* gene in endometrial cancer (EC) is critically important to identify women with a good prognosis and reduce overtreatment. Currently, *POLE* status is determined by DNA sequencing, which can be expensive, relatively time-consuming, and unavailable in hospitals without specialized equipment and personnel. This may hamper the implementation of *POLE*-testing in clinical practice. To overcome this, we developed and validated a rapid, low-cost *POLE* hotspot test by a quantitative polymerase chain reaction (qPCR) assay, *QPOLE*.

**MATERIALS AND METHODS:**

Primer and fluorescence-labeled 5′-nuclease probe sequences of the 11 established pathogenic *POLE* mutations were designed. Three assays, *QPOLE*-frequent for the most common mutations and *QPOLE*-rare-1 and QPOLE-rare-2 for the rare variants, were developed and optimized using DNA extracted from formalin-fixed paraffin-embedded tumor tissues. The simplicity of the design enables *POLE* status assessment within 4-6 hours after DNA isolation. An interlaboratory external validation study was performed to determine the practical feasibility of this assay.

**RESULTS:**

Cutoffs for *POLE* wild-type, *POLE*-mutant, equivocal, and failed results were predefined on the basis of a subset of *POLE* mutants and *POLE* wild-types for the internal and external validation. For equivocal cases, additional DNA sequencing is recommended. Performance in 282 EC cases, of which 99 were *POLE*-mutated, demonstrated an overall accuracy of 98.6% (95% CI, 97.2 to 99.9), a sensitivity of 95.2% (95% CI, 90.7 to 99.8), and a specificity of 100%. After DNA sequencing of 8.8% equivocal cases, the final sensitivity and specificity were 96.0% (95% CI, 92.1 to 99.8) and 100%. External validation confirmed feasibility and accuracy.

**CONCLUSION:**

*QPOLE* is a qPCR assay that is a quick, simple, and reliable alternative for DNA sequencing. *QPOLE* detects all pathogenic variants in the exonuclease domain of the *POLE* gene. *QPOLE* will make low-cost *POLE*-testing available for all women with EC around the globe.

## INTRODUCTION

In 2020, over 417,000 women were diagnosed with endometrial cancer (EC) worldwide, making it the sixth most common cancer in women.^1^ Its incidence has drastically increased in the past few decades, mainly because of an increasing prevalence of obesity and life expectancy.^1,2^ The majority of patients are adequately treated with surgery alone, but still many are recommended to receive adjuvant treatment.^3,4^

**CONTEXT**

**Key Objective**
Molecular classification is essential for prognosis and selection of the most effective treatment for women with endometrial cancer (EC), but how can we provide access to low-cost *POLE* testing for patients around the globe?
**Knowledge Generated**
We developed the *QPOLE* assay, which is a quick, simple, and reliable alternative for DNA sequencing. Performance of *QPOLE* in 282 EC cases, of which 99 *POLE*-mutated, demonstrated an overall accuracy of 98.6% and a final sensitivity and specificity of 96.0% and 100%.
**Relevance**
In comparison with next-generation sequencing, *QPOLE* is a low-cost and quick alternative, which can easily be implemented in pathology laboratories throughout the world, including those with limited resources.

The introduction of the EC molecular classification algorithm, on the basis of four distinct molecular subgroups, has enabled more accurate diagnosis and is used to tailor disease management. One of these molecular subgroups, which accounts for 8%-10% of all EC, consists of ultra-mutated ECs characterized by 11 pathogenic variants in the exonuclease domain (EDM) of DNA polymerase epsilon, *POLE*mut EC. The 2020 WHO classification regards these as a distinct and clinically relevant entity.^5^ These tumors are associated with an excellent clinical prognosis, with a very low risk of relapse, which is independent of histotype and grade and is hypothesized to be of an immunogenic nature.^6-12^ Assessment of *POLE* status in EC is strongly encouraged since women with *POLE*mut EC are effectively cured by surgery alone and are recommended to no or de-escalated adjuvant therapy by the European Society of Gynaecological/European Society Radiation Oncology/European Society of Pathology and European Society for Medical Oncology guidelines.^3-5,7^

Twelve pathogenic somatic missense mutations across 11 loci within exons 9, 11, 13, and 14 of the *POLE* EDM have been internationally recognized to qualify as *POLE*mut EC.^6^ Over 95% of *POLE*mut EC harbors a mutation in P286R, S297F, V411L-T/C, A456P or S459F, whereas only about 4.4% exhibits a mutation in the remaining domains (M295R, F367S, D368Y, L424I, P436R, and M444K).^6,7^ Only these pathogenic driver mutations are associated with an excellent clinical outcome.^6,7^ Currently, no evidence is available if differentiating between the 11 pathogenic *POLE* variants is of clinical significance.

*POLE* status is most often assessed by DNA sequencing methods, such as Sanger or next-generation sequencing (NGS) in the absence of any immunohistochemical (IHC) markers. These techniques are not widely available in hospitals around the world. In addition, they are expensive and time-consuming since equipment and highly trained personnel are required for correct interpretation and as such can take up to 1-2 weeks after surgery before results are available.^13^ Consequently, *POLE*-testing is currently unavailable for most patients with EC, both in high-income as well as middle- and low-income countries.^14^ As a result, women with *POLE*mut EC are overtreated, leading to treatment-related morbidities affecting quality of life and unnecessary costs for radiotherapy and/or chemotherapy.^15,16^

Lack of access to *POLE*-testing hampers implementation of the molecular classification of EC in routine clinical practice worldwide. Although assessment of mismatch repair deficiency (MMRd) and p53 abnormalities by IHC is increasingly performed, assessment of *POLE* status is required for correct molecular classification and allocation of adjuvant therapy.^17^ Finally, a cheap, accessible, and rapid assay, which enables assessment of *POLE* status on tumor material obtained by biopsy or hysterectomy, will aid in counseling and decisions on lymphadenectomy and (neo)adjuvant therapy. Here, we present a quick, simple, and low-cost quantitative genotyping polymerase chain reaction (qPCR) assay for the 11 pathogenic variants in *POLE*, *QPOLE*.

## MATERIALS AND METHODS

### Sample Selection

A sample size calculation was performed to determine the number of required samples to estimate the accuracy of *QPOLE* with 95% CI and an error margin of not more than 10%, which indicated that 73 *POLE*mut cases would be needed.

**FIG 1:**
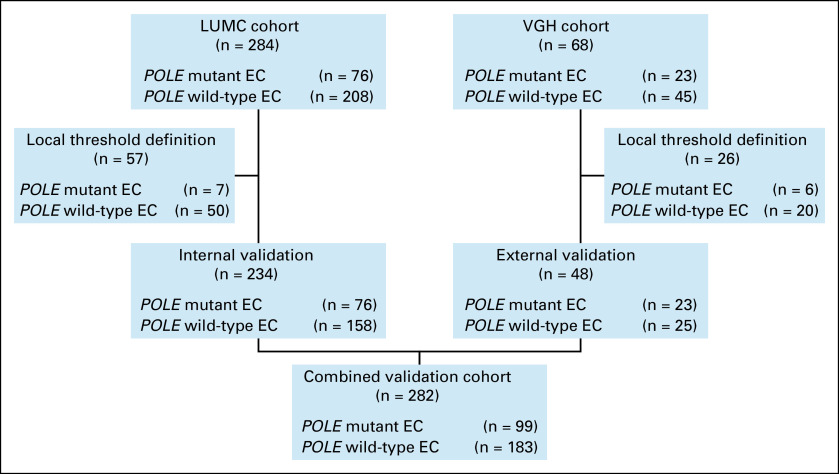
Flowchart of the combined validation cohort. EC, endometrial cancer; LUMC, Leiden University Medical Center; VGH, Vancouver General Hospital.

DNA extracted from formalin-fixed paraffin-embedded (FFPE) tumor tissues from patients diagnosed between 1988 and 2021 was selected from the Leiden University Medical Center EC tumor tissue biobank. DNA isolation was performed between 2017 and 2022.^18^ Of all samples *POLE*mut status was assessed by NGS (AmpliSeq Cancer Hotspot Panel, including the *POLE* EDM [Thermo Fisher Scientific, Waltham, MA]). Cases with a pathogenic variant allele frequency (VAF) of ≥10% were regarded as *POLE*mut. Only cases with a DNA concentration of >5 ng/μL were selected. A total of 284 cases were available, of which 277 were hysterectomies and seven were curetting specimens. Of these, 76 cases harbored a pathogenic *POLE* mutation, whereas 208 were *POLE* wild-type (*POLE*wt; see Figure 1).

To evaluate the transferability of *QPOLE*, an external validation was performed in collaboration with Vancouver General Hospital and Surrey Memorial Hospital, based in Vancouver and Surrey, British Columbia, Canada. A total of 68 cases were available (23 *POLE*mut and 45 *POLE*wt), of which 67 were hysterectomy specimens and additionally, one case was available as biopsy (see Figure 1). FFPE tumor blocks were obtained from patients diagnosed between 2005 and 2017, whose DNA was extracted between 2019 and 2021 using the QIAamp FFPE tissue kit (QIAGEN, Hilden, Germany) or the Qiagen GeneRead DNA FFPE kit (QIAGEN). *POLE* status was determined by NGS, and only cases with a VAF of ≥10% and a DNA concentration of ≥5 ng/μL were selected.

Molecular subgroup assignment of *POLE*wt cases was performed by IHC for MMRd and abnormal p53 expression according to the WHO 2020 classification for both validation phases.^5^

### *QPOLE* Assay Design

We designed a probe-based qPCR assay since this modality is cheap, quick, sensitive, and user-friendly. In short, primer pairs for the four exons within the EDM of *POLE* and Affinity Plus probes consisting of locked nucleic acids with a 5′ fluorophore (FAM dye for *POLE*wt alleles and HEX or SUN dye for *POLE*mut alleles) and a 3′ Iowa Black Quencher were designed and developed by Integrated DNA Technologies (Coralville, IA), Table 1. In the case of a homozygous sample, predominantly FAM relative fluorescence units (RFUs) would be detected, whereas in a heterozygous sample, higher HEX/SUN RFUs would be observed. No cross-reactivities were detected for primer and probe sequences in the University of California Santa Cruz Genome Browser or while performing calibration studies.^19^

**TABLE 1:**
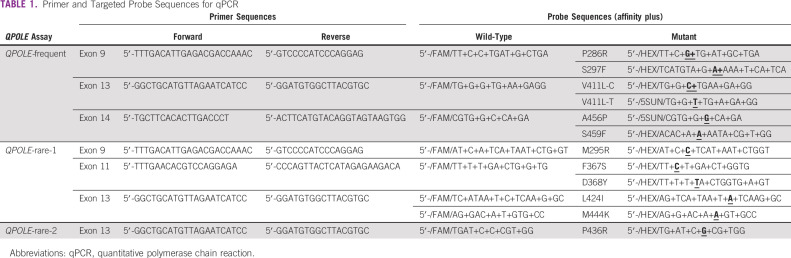
Primer and Targeted Probe Sequences for qPCR

Experiments were performed to reduce the number of PCR reactions from 12 singleplex reactions to a limited number of reactions by evaluating grouping of the respective primers and probes into different mixes. This resulted in three assays: (1) *QPOLE*-frequent including all five frequently occurring mutations, (2) *QPOLE*-rare-1 for five of six rare variants, and (3) *QPOLE*-rare-2 for detection of P436R. All reactions were performed with 10 ng of DNA in a final volume of 10 µL, consisting of PCR mixes, in a Bio-Rad hard shell 96-well PCR plate (Bio-Rad Laboratories, Hercules, CA). Amplification and detection of the PCR products were followed on a C1000 Thermal Cycler Bio-Rad, CFX96-well Real-Time PCR machine (Bio-Rad Laboratories) under the following conditions: 95.0°C for 3 minutes; followed by 95.0°C for 10 seconds, 58.0°C for 1 minute, and 72.0°C for 30 seconds for 40 cycles (total time ±100 minutes). Analyses were performed using the Bio-Rad Maestro program (Bio-Rad Laboratories). A more detailed description is given in the Data Supplement.

Prevalidation studies were performed to calibrate and verify the performance of *QPOLE*, and to determine predefined thresholds for sample calling for the two validation phases on different CFX96-well real-time PCR machines, in Leiden and Surrey, respectively. The following types of results were predefined: wild-type, mutant, failed, and equivocal. Thresholds were based on lowest FAM RFU for failed and lowest HEX/SUN RFU for *POLE*mut samples and highest HEX/SUN RFU for *POLE*wt samples. A range of uncertainty was added between wild-type and mutant thresholds to function as a safety margin. Samples within this range were specified as equivocal, and additional DNA sequencing (Sanger or NGS) is recommended for these cases. Retesting of *QPOLE* is recommended for failed cases. Thresholds during the internal validation phase were defined using 50 *POLE*wt and seven *POLE*mut cases (P286R [n = 2], V411L-T [n = 2], and V411L-C [n = 1] for *QPOLE-*frequent and L424I and M444K for *QPOLE*-rare-1), Data Supplement. For the external validation phase, 20 *POLE*wt and six *POLE*mut (P286R, V411L-T, and V411L-C for *QPOLE*-frequent and M295R, F367S, and D368Y for *QPOLE*-rare-1) were used (Data Supplement).

### Validation Studies

During the internal and external validation phases, a total of 234 EC and 48 EC cases, respectively, were tested to calculate the accuracy, sensitivity, and specificity of *QPOLE*. All samples were tested in duplicate. In case two samples of one patient had different types of results, the following algorithm was applied: (1) mutant and equivocal were deemed mutant; (2) wild-type and equivocal were considered equivocal and assigned to additional DNA sequencing; (3) wild-type and mutant were regarded as failed and assigned to retesting with *QPOLE*; (4) if one of two samples failed, assignment was performed on the basis of the result of the other sample, unless the duplicate was classified as mutant. In that case, additional DNA sequencing was recommended, Table 2.

**TABLE 2:**
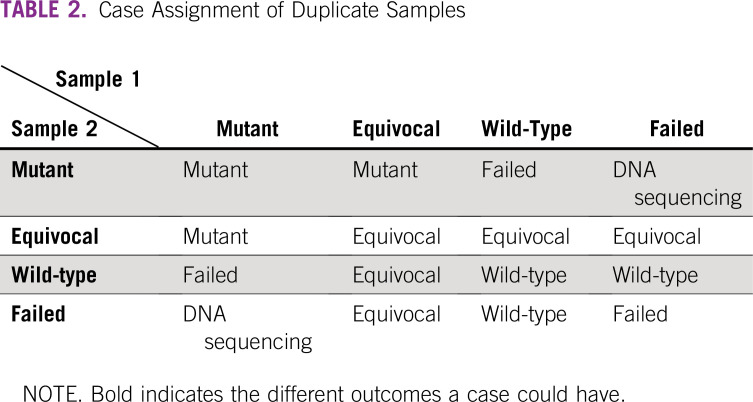
Case Assignment of Duplicate Samples

## RESULTS

### Sample Characteristics

The majority of *POLE*mut cases of the internal validation cohort (63 of 76) harbored a mutation in P286R or V411L-C/T. Other *POLE* variants were S297F, L424I, M444K, A456P, and S459F. VAFs for *POLE*mut cases varied between 15.0% and 81.6%. The majority of *POLE*wt samples had no specific molecular profile (NSMP) EC of 43.0%, followed by MMRd EC of 19.1%, and p53-abnormal EC of 11.9%. Over 89% were of endometrioid-type, mostly low-grade tumors, Table 3. The DNA concentration for all cases varied between 6.7 and 132 ng/μL.

**TABLE 3:**
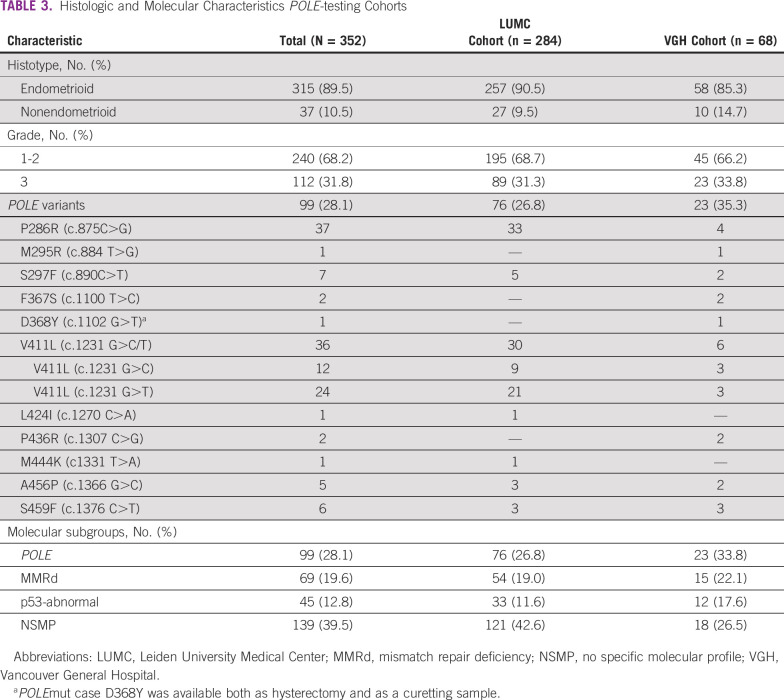
Histologic and Molecular Characteristics *POLE*-testing Cohorts

In the external validation, of the 48 hysterectomy samples (including one case as additional biopsy), VAFs for the 23 *POLE*mut cases varied between 12.5% and 38.68%. Also here, the majority were low-grade endometrioid tumors, Table 3. Of the *POLE*wt tumors, 26.5% were NSMP, whereas only 17.1% and 21.4% were p53-abnormal and MMRd, respectively. The DNA concentration varied between 5.26 and 10.3 ng/μL.

### Validation

#### Internal validation phase.

**FIG 2:**
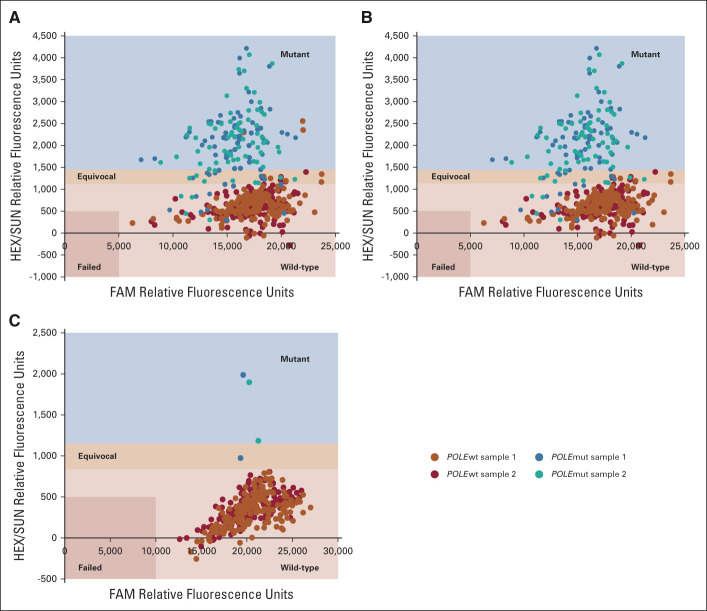
Internal validation of *QPOLE*-frequent and *QPOLE*-rare-1. (A) and (B) show the allelic discrimination graph of *QPOLE*-frequent. (A) Three wild-type blue and red dots can be distinguished in the orange or teal mutant quadrant of *QPOLE*-frequent, whereas their duplicates were within the wild-type quadrant. (B) After reperformance of the assay, all the duplicates of these three cases fell within the blue wild-type quadrant. (C) shows the allelic discrimination graph of *QPOLE*-rare-1.

Of 234 available EC cases, all 158 *POLE*wt cases tested by *QPOLE*-frequent fell below the mutant threshold. Of 74 cases with frequently occurring *POLE* variants, 59 cases were correctly called, 11 cases fell within the equivocal range, four cases were misclassified, and three failed (1.3%), Figure 2A. Failed cases were retested with *QPOLE*-frequent and subsequently classified as *POLE*wt, Figure 2B. Quality PCR analyses showed that the DNA of the four misclassified cases was heavily fragmented. *QPOLE*-frequent had a crude sensitivity of 93.7% (95% CI, 87.7 to 99.7) and a specificity of 100%. Confirmatory DNA sequencing for the 11 cases (4.7%) within the equivocal range yielded a sensitivity of 94.6% (95% CI, 89.4 to 99.7).

*QPOLE*-rare-1 was tested on the same 158 *POLE*wt cases and two rare *POLE* variants (L424I and M444K). None of the *POLE*wt cases were misclassified or failed, and only one of the mutated samples fell within the equivocal range, Figure 2C. Thus a sensitivity and specificity of both 100% could be obtained for *QPOLE*-rare-1 as well as *QPOLE*-rare-1 combined with DNA sequencing.

Taking equivocal cases, which needed additional DNA sequencing into account, combining *QPOLE*-frequent and *QPOLE-*rare-1 demonstrated an accuracy of 98.2% (95% CI, 96.5 to 99.9), with a sensitivity of 93.8% (95% CI, 88.0 to 99.7) and a specificity of 100%. Combining *QPOLE*-frequent and *QPOLE*-rare-1 with DNA sequencing only for equivocal results (8.1%) showed a final accuracy of 98.3% (95% CI, 96.7 to 99.9) and a sensitivity of 94.7% (95% CI, 89.7 to 99.8), while maintaining 100% specificity.

*QPOLE*-rare-2 was not tested in the internal validation phase because the rare pathogenic variant P436R was not present in the cohort for internal testing.

#### External validation.

**FIG 3:**
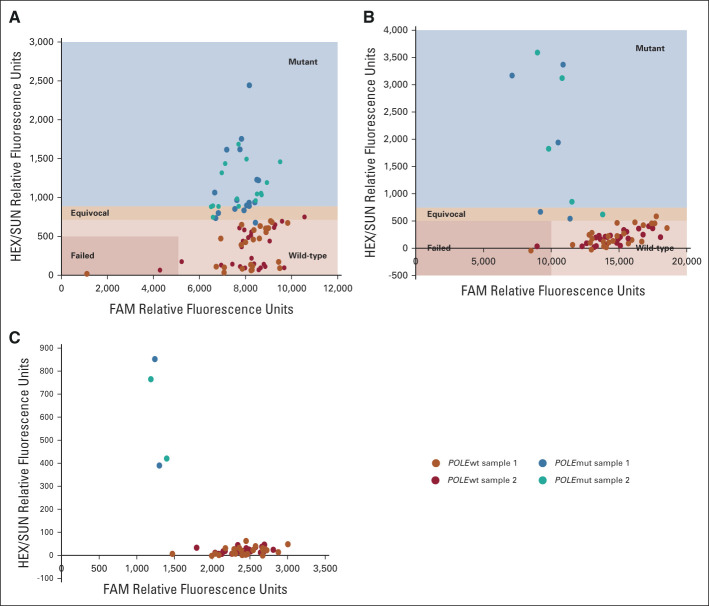
External validation of *QPOLE*-frequent, *QPOLE*-rare-1, and *QPOLE*-rare-2. (A) and (B) show the allelic discrimination graph of *QPOLE*-frequent (A) and *QPOLE-*rare-1 (B). (C) shows the allelic discrimination graph of *QPOLE*-rare-2; case assignment was performed on the basis of visual discrimination of the *POLE*mut EC samples versus the *POLE*wt EC samples. EC, endometrial cancer.

*QPOLE* was run on 48 cases, of which 23 harbored a *POLE* mutation; 17 were frequently occurring variants, and six were rare variants. Of the 17 *POLE*mut cases tested by *QPOLE*-frequent, 14 were assigned correctly, two fell within the equivocal range, and one failed case was accurately diagnosed as mutant by *QPOLE*-frequent after retesting. Of the 25 wild-type cases, 20 were tested as wild-type, whereas three were equivocal, and two failed cases were correctly classified after retesting (Fig 3A). Hence, both sensitivity and specificity were 100% for *QPOLE*-frequent. The remaining six *POLE* cases were tested by either *QPOLE*-rare-1 or *QPOLE*-rare-2. All *POLE*mut cases were correctly classified by *QPOLE-*rare-1, and of all wild-type samples, only one case fell within the equivocal range (Fig 3B). Two P436R *POLE* cases were assessed using *QPOLE*-rare-2 and were clearly separated from all *POLE*wt cases (Fig 3C), resulting in a sensitivity and specificity of 100% for both *QPOLE*-rare assays (Fig 3C). Taking equivocal cases (10.4%) into account, which needed additional DNA sequencing, the overall accuracy both with and without DNA sequencing was 100%, with a sensitivity of 100% and a specificity of 100%.

#### Combined analysis of the internal and external validation.

The combined validation cohort consisted of 282 unique EC cases, of which 99 were *POLE*mut. Only four *POLE*mut cases were not detected, and 15 *POLE*mut cases (5.3%) were detected after an equivocal result by DNA sequencing. The overall final accuracy, calculated by DNA sequencing for equivocal cases, sensitivity, and specificity of *QPOLE* on the basis of these two phases were 98.6% (95% CI, 97.2 to 99.9), 95.2% (95% CI, 90.7 to 99.8), and 100%, respectively. If DNA sequencing was performed only for equivocal results (8.8%) after upfront *QPOLE* testing, the overall final sensitivity and specificity were 96.0% (95% CI, 92.1 to 99.8) and 100%, respectively (Table 4 and Data Supplement).

**TABLE 4:**
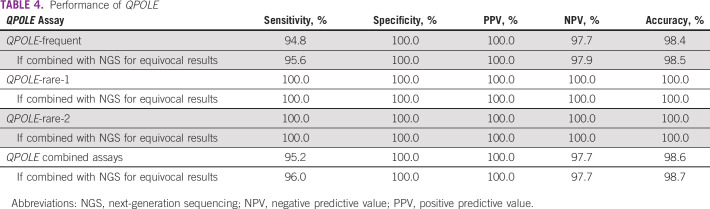
Performance of *QPOLE*

## DISCUSSION

In this study, we presented a laboratory-developed, quick, accurate, and low-cost alternative to DNA sequencing of all 11 pathogenic *POLE* mutations, *QPOLE*. Our method ensures that assessment of *POLE* status can be performed in any laboratory around the world, which has a qPCR machine and the capacity to isolate DNA from FFPE tissues. qPCR methods are cheap and widely available, have proven clinical reliability, and are used in first line of diagnostics in many countries today.^16^ *QPOLE* significantly reduces the turnaround time to only 4-6 hours compared with up to 1 or more weeks for conventional NGS. After establishment of local thresholds for sample calling, *QPOLE* has shown excellent performance in internal and external validation, and implementation in another laboratory was simple. Even when using DNA from older FFPE blocks, comparing *QPOLE* with golden standard NGS showed an accuracy of 98.6% (95% CI, 97.2 to 99.9) and a sensitivity and specificity of 96.0% (95% CI, 92.1 to 99.8) and 100%, respectively.

Two other *POLE*-testing alternatives have been recently published, a SNaPshot and a droplet digital PCR assay.^20,21^ These alternatives are quick and potentially cheap as well; however, both techniques require specific equipment to perform the analyses, such as a Qx200 Droplet Digital PCR system (Bio-Rad, Hercules, CA), Eppendorf thermocycler (Proflex PCR system, Life Technologies, Carlsbad, CA), or ABI 3500xL (Applied Biosystems, Foster City, CA). For comparison, *QPOLE* can be run on any qPCR device as long as the commonly used HEX and FAM channels have been calibrated. The other two methods reported a sensitivity and specificity of 100%.^20,21^ It is important to note that these assays have only been validated on 11 and 26 *POLE*mut EC cases, respectively, and included only most occurring *POLE* variants and none of the rare *POLE* variants. In a follow-up study, the SNaPshot assay was tested on 15 *POLE*mut EC cases, of which only two harbored a rare pathogenic variant.^22^ For comparison, *QPOLE* has been tested on 91 *POLE*mut EC cases including all frequently occurring *POLE* variants and eight *POLE*mut EC cases harboring all rare *POLE* variants. As such, *QPOLE* is the only assay that has been validated on all pathogenic *POLE* variants with a sufficiently large sample size to provide accurate estimates of test performance. Finally, no external validation was performed for the other *POLE* tests. Therefore, it is currently not known whether these assays are directly transferable to any other laboratories. Adaption of *QPOLE* on the QuantStudio real-time PCR system (Thermo Fisher Scientific, San Fransisco, CA) is currently ongoing.

In this study, only four cases (1.4%) were misclassified, which may be caused by a high fragmentation of the DNA in these samples. Still, a sensitivity of 96.0% (95% CI, 92.1 to 99.8) was observed, a concordance between *QPOLE* and golden standard NGS, which is in line with other surrogate marker comparison studies within EC.^23-25^ IHC to assess MMR and p53 status is currently the preferred approach within the WHO 2020 diagnostic algorithm of EC.^3,5^ Concordance between MMR status by IHC and the microsatellite instability assay is 93.3% (sensitivity of 88.5%, specificity of 95.2%).^23^ As for the presence of *TP53* mutations by DNA sequencing and abnormal p53 staining pattern on IHC, accuracies between 94.5% and 95.1% have been reported (sensitivities of 95.0%-97.7% and specificities of 94.1%-94.3%).^24,25^

We aimed to combine all 12 probes for *POLE* variants in as few qPCR reactions as possible to optimize cost efficacy and feasibility of implementation in routine clinical diagnostics. We were able to bring the number of qPCR reactions down from 12 singleplex reactions to two multiplex reactions and one singleplex reaction. If novel pathogenetic *POLE* variants will be identified in the future, these could be added to *QPOLE*-rare-2. As such, *QPOLE* is flexible and future proof. Preferably, the three *QPOLE* assays are run simultaneously; however, if resources are limited, they can be performed in a stepped approach, starting with *QPOLE*-frequent and only performing *QPOLE*-rare-1 and *QPOLE*-rare-2 if negative. An even more economical option is to only use *QPOLE-*frequent, which will still detect 95% of pathogenic *POLE* mutations.^6,7^ If DNA sequencing for equivocal test results, which occurs in about 8.8% of cases, is not feasible, *QPOLE* still has an excellent performance of 95.2% (95% CI, 90.7 to 99.8).

Despite the rarity of some pathogenic variants, we composed a validation set including seven cases, containing at least one case of each rare variant. Using these cases to determine the thresholds and validation was unavoidable. An essential step during the setup of *QPOLE* in a new laboratory is determination of local thresholds. As such, it is possible that initial thresholds are broader as a safety measurement, especially for equivocal, to correct for the range of uncertainty. Another limitation is that *QPOLE* is a laboratory-developed test and might need regulatory clearance and approval depending on local regulations, before it can be implemented in daily clinical practice.

Without taking DNA isolation and preparation into consideration, the simplicity of *QPOLE* enables *POLE* status assessment within 4-6 hours. Furthermore, *QPOLE* does not require interpretation by a molecular biologist, as opposed to NGS. As a result, *QPOLE* is substantially faster and cheaper in comparison with most current DNA sequencing techniques. Furthermore, *QPOLE* results could be used during preoperative counseling on lymphadenectomy.

*QPOLE* will not only enable *POLE*-testing around the world but also yield less morbidity through treatment de-escalation and a reduction in health care–related costs. Cost-effectiveness of the molecular classification in advanced EC has been predicted by a modeling study and is currently under investigation in the PORTEC-4a trial.^26,27^ Additional savings of replacing DNA sequencing with *QPOLE* will make the assessment of the molecular classification even more cost-effective.

In conclusion, the laboratory-developed *QPOLE* test is a quick, low-cost, and accurate *POLE*-hotspot testing alternative for the detection of pathogenic *POLE* mutations, enabling implementation in clinical practice with consequences for clinical management.
